# Industrial functional safety assessment for WSN using QoS metrics

**DOI:** 10.1016/j.heliyon.2022.e11255

**Published:** 2022-11-07

**Authors:** Sivasubramanian Srinivasan, T.K. Ramesh, Roberto Paccapeli, Luca Fanucci

**Affiliations:** a Department of Electronics and Communication Engineering, Amrita School of Engineering, Bengaluru, Amrita Vishwa Vidyapeetham, India; b Red Hat, Italy; c Department of Information Engineering, University of Pisa, Via G Caruso, 16-56122, Pisa, Italy

**Keywords:** Industrial functional safety, Message defenses, Message threats, QoS metrics, QoS guarantees, Safety integrity level, Wireless sensor networks

## Abstract

Wireless Sensor Networks are increasingly getting deployed for the safety use cases in industrial applications. While several research papers discuss about the Quality & Reliability improvement techniques in WSN systems to achieve minimal delay, higher node life, optimal routing etc., very limited work is witnessed on assessment of safety integrity levels of WSN systems. In this paper we tried to bridge this gap by bringing out a QoS metric-based safety integrity assessment for the end-to-end industrial Wireless Sensor Network (WSN) system. To identify relevant QoS metrics for monitoring the safety integrity levels, we also bring out a 4-step mapping methodology to link the QoS metrics and communication defenses/safety mechanisms. This mapping approach is expected to serve the network safety design engineers. Finally, a simulation case example is discussed to illustrate safety integrity assessment and we conclude by bringing out future research opportunities to improve safety integrity levels of industrial WSN systems.

## 1 Introduction

Wireless communication is becoming an essential part of human life. However, due to its vulnerability to noise interference and security attacks which could result in data corruption, latency, low throughput etc., deployment of wireless communication is traditionally considered only for the non-safety applications in the industries. For example, monitoring of non-safety process variables, supervisory functions etc., Conservatively, in safety applications such as process control, actuation of emergency systems etc., wireless communication is deployed only as a backup option [1]. It may however be noted that deploying wireless communication for safety applications is not forbidden [2]. It is observed in [3] that the value proposition for the Industrial WSN (IWSN) has increased due to its deployment in closed loop control and safety systems. Further, WSN is an inseparable part of industrial IoT and the concept of Industry 4.0 is characterized by flexible manufacturing, collaborative robotics supported by reliable communication between sensors, actuation systems and computing elements [4], [5]. Hence, we are of the view that WSN systems can be deployed for safety applications after evaluating its end-to-end Safety Integrity Level (SIL) [6]. Here the end-to-end WSN system refers to both hardware as well as the data communication involving the source node, hop path, routers, and destination edge devices. In this paper we bring out an estimation process of SIL for the end-to-end communication system using the QoS metrics. As regards the SIL for the hardware elements, reader can refer to the techniques discussed in Annexure-D of [7]. Since QoS metrics may vary due to external noise interference and channel noise performance, the variation in safety integrity level needs to be monitored in real-time and statistically interpreted for its acceptability. It is expected that the said assessment process would serve the industrial WSN safety design and maintenance engineers. To illustrate the approach of SIL estimation for the end-to-end system, we introduce a mapping technique that is expected to help the safety design engineers/network maintenance engineers to identify relevant QoS metrics for monitoring.

This paper is organized as follows. First an overview on industrial safety integrity level is outlined in Section 2. In Section 3, relevance of QoS metrics to functional safety is discussed. We then, provide typical wireless QoS metrics deployed for WSN in Section 4. A 4-step mapping methodology is proposed in Section 5 relating the QoS metrics and communication defenses which can help to identify relevant QoS metrics for monitoring the SIL of the end-to-end system. In Section 6, we demonstrate the safety assessment approach with two simulation case examples. Finally, in Section 7 we bring out the conclusions and future research opportunities to augment functional safety in end-to-end WSN using QoS metrics.

## 2 Overview on industrial functional safety

The meaning of safety as defined in the English dictionary is “the condition of being protected from or unlikely to cause danger, risk, or injury.” [8] The industrial safety is a broad area of safety, and it refers to the workplace safety. The workplace covers specific safety issues related to the site and process encompassing areas such as material safety, fire safety, electrical safety, environmental safety etc., The potential hazards associated with safety areas could cause loss of life, loss of property or huge adverse impact on environment. Hence, safety hazards are identified proactively, and measures are engineered to prevent or minimize the loss. The Functional Safety (FS) is another part of safety which is associated with systems or equipment meant to respond to dangerous situations to ensure safety [9]. The aim of functional safety implementation is to bring down the risk associated with potentially dangerous conditions that can result into an accident harming someone or destroy something.

The basic functional safety standard IEC-61508 provides guidelines for development of safety systems [10] and addresses Electrical, Electronic, or programmable electronic systems (E/E/PE) that carryout the safety functions (SF). The standard defines the safety integrity levels (SIL) for systems that carryout safety functions. Compliance to SIL as defined in IEC-61508 implies that the risk associated with a safety system is at an acceptable level.

There are two major types of faults associated with E/E/PE systems and are a) Random hardware failure of components and b) Systematic faults in the development process. The hardware faults occur due to random causes such as operating stress exceedance, marginally weak parts, random errors due to installation or maintenance actions etc., Such random hardware failures are probabilistic in nature and are expressed as number of failures per unit time referred as failure rate. Typically, failure rates are very low for reliable components or systems and will be of the order of a few failures over multiple million operating hours. Hence for mathematical convenience it is expressed as ‘Failures-In-Time (FIT)’ which refers to number of failures per billion operating hours [10], [11]. The SIL differentiates the level of robustness of systems for meeting the safety objectives. There are 4 levels of SIL for industrial systems denoted as SIL N, where N varies from 1 to 4. A higher value of ‘N’ denotes higher safety integrity i.e., lower probability of failure of the safety functions due to random hardware faults. Depending on the application scenario, the operating duty cycle of the safety systems will vary, and the safety integrity targets differ. When the demand rate of safety systems is less than or equal to a one per year, it is considered as low demand systems whereas if the demand rate is greater than once a year it is a high demand system [12]. It is intuitive that a low demand system has a lower safety target as compared to a high demand system. The SIL-1 target probability of failure for a high and low demand systems are 10^−6^ ≤ p < 10^−5^ and 10^−2^ ≤ p < 10^−1^ respectively. Similarly, for SIL-2 high & low demand systems these values are 10^−6^ ≤ p < 10^−5^ and 10^−2^ ≤ p < 10^−1^ respectively. The quantitative requirements for SIL-3 & 4 can further be referred in section-7 of [10]. As regards the systematic faults, these are caused by development processes that typically do not adopt quality management principles such as requirements traceability, change management, configuration management, Failure analysis and corrective action system etc., Further details on the requirements to mitigate systematic faults could be referred in Annexure B of [11]. The capability of a safety system against systematic faults is referred as ‘SC N’ where N varies from 1 to 4. The systematic capability ‘SC N’ means that the safety integrity SIL N is met by the system against systematic faults. Reader can further refer to Section-3 of [13].

*Data communication threats and defenses*

Loss of data or data corruption are the threats common to both wired and wireless systems, however, the probability of occurrence of such threats differ for both. The common functional safety principles used in transmission of safety-relevant messages are discussed in references [14], [15]. Safety principles refer to safety mechanisms or defenses built into data communication protocol for robustness against data threats. The safety defenses work against specific threats and this mapping is shown in Table 1. For example, the threat of message repetition due to channel noise, can be mitigated by building the defense mechanisms [4] like sequence number, time stamp and time triggered architecture etc... Thus, building the defenses in the protocol enhances the safety of data communication.

**Table 1:**
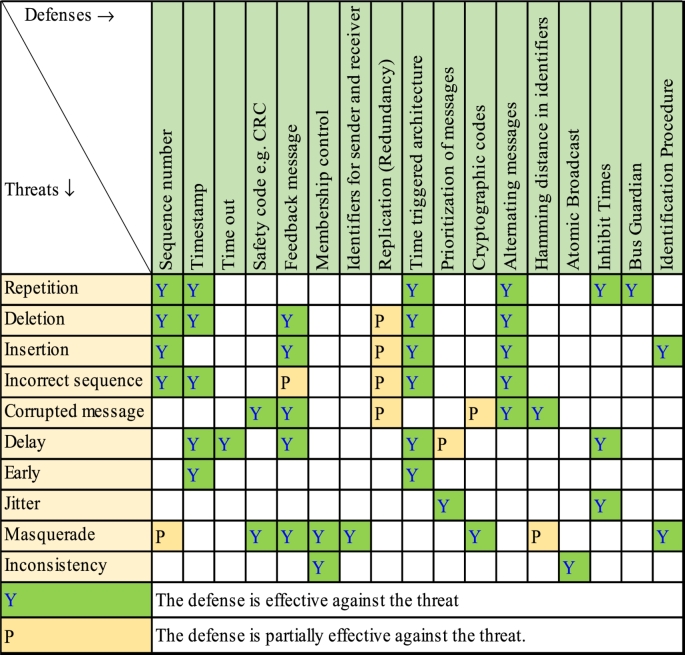
STEP-1 Mapping defenses and message threats.

## 3 QoS metrics and relevance to functional safety

The Quality of service (QoS) metrics of a WSN indicates the ability of the network to meet performance parameters like latency, error rate, throughput, jitter etc., Applications such as multimedia communication, process control systems, health care systems etc., are sensitive to network performance parameters and hence the WSN communication protocols implement specific defense mechanisms to meet QoS metrics.

To meet the QoS objectives of the network, various approaches like network routing schemes, modulation schemes, device clustering etc. are adopted while optimizing power consumption by controlling the sleep states of transmitters & receivers, use of broadcast messages-based protocol etc., Clustering approaches to strengthen the performance of WSN in respect of QoS metrics could be found in [16]. To maintain the QoS of WSN at a level, QoS aware routing algorithms are deployed. A survey of QoS aware routing protocols for the Mobile Ad hoc Networks (MANET) – WSN convergence scenarios in IoT networks is available in [17]. QoS aware routing algorithm for MANETS to balance minimization of the consumed timeslots while providing required timeslots for the nodes in a route having heavier traffic load is discussed in [18].

In the context of Safety, SIL is specified as a probability and depends on underlying random failure causes. Similarly, QoS metrics of an end-to-end WSN is also probabilistic in nature and is influenced by the communication threats that are caused by random phenomena like interference due external noise. Thus, the QoS metrics inherently reflects the ability of an end-to end system to meet both the performance as well as the safety objectives and can thus be leveraged for compliance assessment. The QoS requirements are driven by application objectives. For example, real time applications like vehicle control systems, weapon systems, video streaming for medical procedures, factory automation for process control etc., place high demand on network QoS to have guaranteed delay, loss, jitter, and throughput [19]. Typically, a communication failure in such real time systems could lead to safety critical effects. Hence evaluating the performance quality of communication would assure the integrity of the communication systems deployed for safety functions.

Now let us look at the service guarantees of QoS metrics. The major service guarantees of QoS metrics could be segregated as Guarantee of Loss (GoL), Guarantee of Delay (GoD), Guarantee of Throughput (GoT), and Guarantee of Jitter (GoJ). The guarantee of loss is based on keeping the ratio of data packets lost from sender to receiver below a specified fraction. The guarantee of delay is offered by not exceeding a threshold value of delay while the guarantee of jitter is by maintaining the variation of delay below a threshold. As regards the guarantee of throughput, it is based on keeping the number of data packets transmitted from sender to receiver above a required value.

## 4 QoS metrics in industrial applications

In the following paragraphs typical QoS metrics used for industrial WSN systems and use scenarios are discussed.

• *Successful Detection Rate (SDR) & False Positive Detection Rate (FPDR)*

The SDR and FPDR metrics discussed in [20] will serve for real-time safety monitoring. The SDR and FPDR are respectively expressed as the ratio of number of successful detections to number of anomalous detections and Number of false detections to number of normal measurements, respectively. These QoS parameters are used in the applications where large-scale Networked Industrial Sensing Systems (NISS) are connected and communicating, for example wireless sensor networks, smart grid systems etc.

• *Transmission Efficiency (TE)*

The transmission efficiency indicates the throughput ability of network stations to complete the data transmission in a shortest time. It is directly proportional to number of network stations, probability of transmission by a given network station and the average transmission time available for the station. It is inversely proportional to the time taken to complete transmission of all the bytes in the data sequence [21].

• *Delay Bound (DB)*

The timeliness of delivery of data packets is one of the critical QoS metrics for real-time systems. The channel access delay for a single hop communication and the multi-hop end-to-end (E-2-E) delay is discussed in [22]. The need to tune task scheduling to achieve probabilistic delay targets and minimum bandwidth are highlighted in [23]. An analytical model relating the scheduling time, data traffic load, packet delay and dropped packet rate are developed in [24].

• *Area Spectral Efficiency (ASE)*

The QoS constrained Area Spectral Efficiency is a metric introduced in [25] to assess the performance of downlink in Ultra-Reliable Low Latency Communications (URLLC). This metric has been used to assess the performance of the communication link under conditions of low E-2-E delay typically less than 4 msec in cloud connectivity, industrial automation links and wireless links in automobiles, etc., and about 1 msec in case of rapidly moving devices applicable to the 5^th^ generation era and reliability exceeding 99.999% for 32 bytes [26].

• *Packet Delivery Ratio (PDR)*

Data packets must be reliably delivered to the destination nodes of a WSN utilizing minimal power consumption. Algorithms for efficient geographic opportunistic routing keeping the E-2-E reliability, delay, and energy efficiency as constraints for a proposed route are discussed in [27]. Reliable reactive routing protocols for enhanced packet delivery with energy efficiency are presented in [28].

• *Signal to Noise Ratio*

The Signal-to-Noise Ratio (SNR) is one of the metrics that evaluate the quality of the link and characterizes both the non-linear and non-stationary random features [29] and is expressed as the ratio of signal power input to a receiver and the noise power of the receiver hardware [30]. The link quality can randomly vary due to atmospheric conditions, obstructions, path length variations between source and destination nodes, etc. The SNR decides the QoS metric of Packet Reception Ratio (PRR).

• *End-to-End Data Delivery Reliability (DDR)*

The E-2-E Data Delivery Reliability is a QoS metric that is used to estimate and optimize WSN reliability [31]. The E-2-E-DDR model is a function that maps QoS parameters such as Packet Reception Ratio (PRR) and Signal to Noise Ratio (SNR) [29]. Further discussion on E-2-E acknowledgment method to improve network reliability can be referred in [32]. The QoS metrics discussed above are summarized in Table 2. The summary provides the reference publication, the QoS metric and the characteristic guarantees.

**Table 2:** QOS metrics used for industrial WSN.

Survey Reference	QoS metrics	Guarantees
Real-Time Detection (General Anomaly Detection-GAD) [20]	Successful Detection Rate (SDR)	GoL, GoT
	False Positive Detection Rate (FPDR)	GoL, GoT

Multi-Polling controlled access (MPCA) [21]	Transmission Efficiency (TE)	GoT
	Channel Throughput / Packet Loss / Packet Delay / Peak Signal to Noise Ratio	

Group based M2M communications [33]	Delay Bound (DB)	GoD, GoJ

Area Spectral Efficiency (ASE) as a new QoS metric [25]	• QoS constrained area spectral efficiency	GoL, GoD
	• E-2-E delay	

Energy-Efficient Reliable Routing [34]	Packet Delivery Ratio (PDR) for n-hops of a path	GoL, GoT

Probability-guaranteed limits on the packet reception ratio (PRR) [29]	Packet Reception Ratio (PRR)	GoL, GoT

E-2-E data delivery reliability model (E-2-E-DDR) at network-level [31]	E-2-E Data Delivery Reliability	GoL, GoT

## 5 Mapping - QoS metrics, threats, and defenses

**Figure 1:**
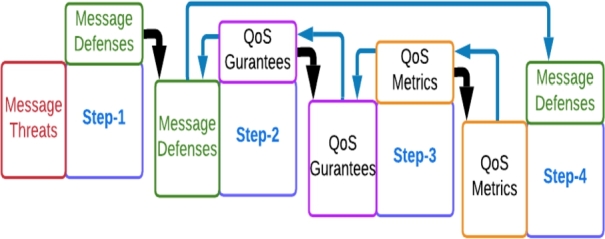
Mapping QoS metrics and message defenses.

In this section we will discuss a mapping methodology to identify QoS metrics that are relevant for monitoring the safety integrity level of a WSN based on the message level defense(s). The proposed mapping between message defenses and QoS metrics are achieved in 4 steps as shown in Fig. 1. The Step-1 starts by mapping the message threats Vs message/architectural defenses [35], [36] as explained in section 2 and shown in Table 1.

Here the row factors are the message threats, and the column factors are message/architectural defenses. The mapping indicates if a defense is effective to mitigate a threat. The Step-2 involves mapping of these defenses and QoS guarantees as shown in Table 3. The objective here is to assess which message defense would be effective for the QoS guarantee(s). For example, ‘time stamp’ as a defense would offer Guarantee of delay and guarantee of jitter. Similarly, the ‘CRC code’ as a defense, would offer guarantee of data loss/data corruption. If a defense is partially effective, then it is marked as ‘PY’ indicating ‘Partially Yes’. This means the specific defense may indirectly offer the QoS guarantee. In Step-3 the QoS guarantees and the QoS metrics are mapped as shown in Table 4. In Step-4, the message defenses relevant for a QoS metric are identified and mapped in Table 5. To identify the defenses to be mapped for a given QoS metric in step-4, we travel back to step-3 and step-2 by following the blue colored arrow lines shown in Fig. 1 to determine the message defenses corresponding to a given QoS metric. Thus, in this step, QoS metrics that offer a relevant message defense to improve the integrity levels can be identified. The final mapping between QoS metrics Vs Defenses is shown in Table 5.

**Table 3:**
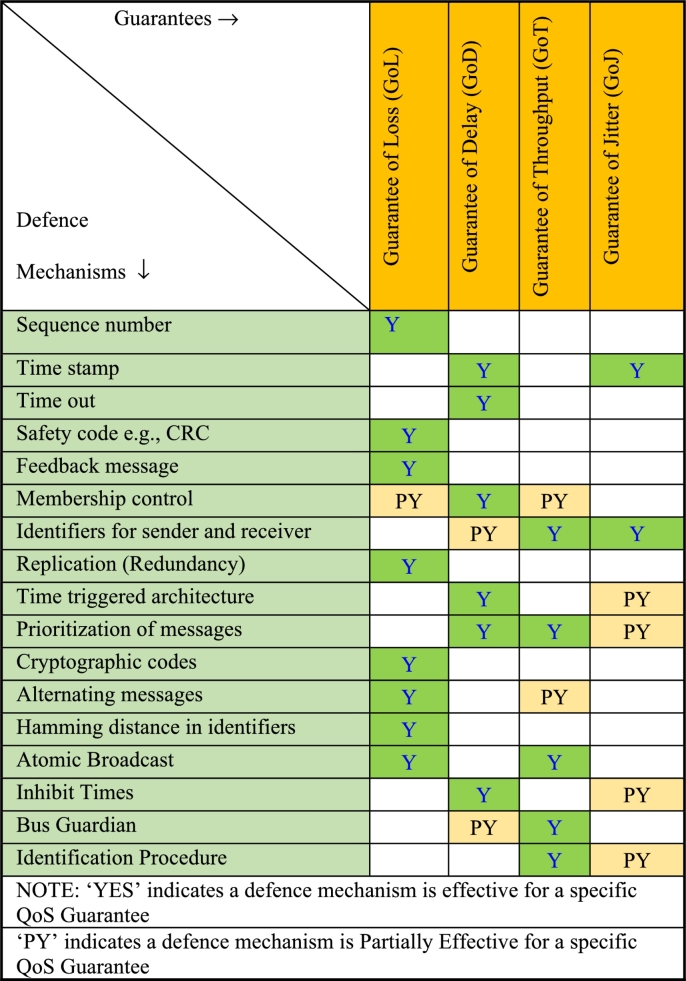
STEP-2_Mapping defense mechanisms and QoS guarantees.

**Table 4:**
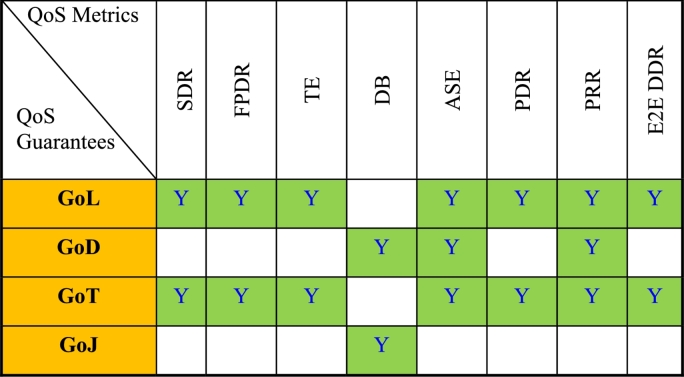
Step-3_Mapping QoS guarantees and QoS metrics.

**Table 5:**
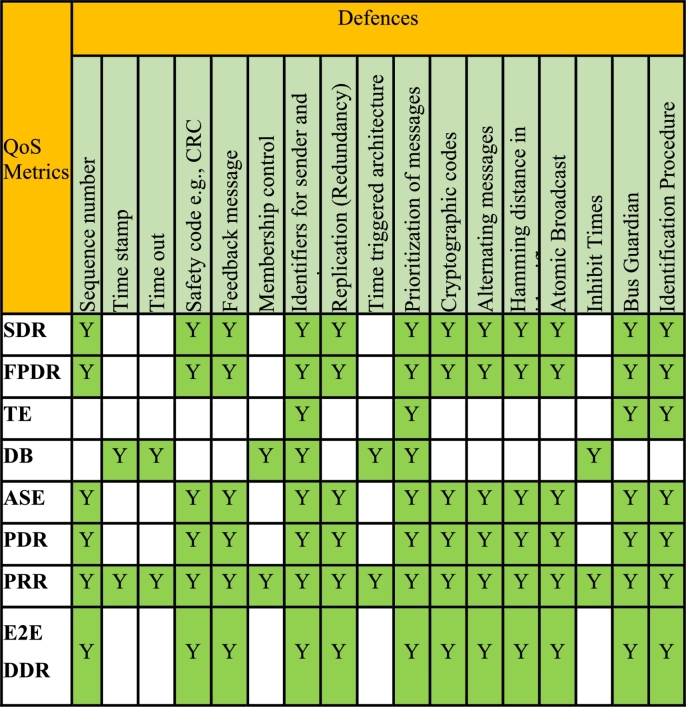
Step-4_Mapping QoS metrics and defenses.

## 6 Simulation example and results

To illustrate use of QoS for real time safety assessment in industrial WSN, we choose the Successful Detection Rate (SDR) metric which could be defined as shown in equation (1).

(1) $$\mathrm{SDR}=\left(\frac{\mathrm{Successful}\;\mathrm{Detections}}{\mathrm{Successful}\;\mathrm{Detections}+\mathrm{Anamolous}\;\mathrm{Detections}}\right)$$

The SDR is the ratio of successful detection of data packets to total data packets detected at the destination node. The anomalies can typically be detected either by a centralized controller or by any localized sensing device i.e., by the nodes. From viewpoint of safety, higher SDR implies lower data drop out and higher probability of meeting the process safety time requirements of safety critical systems. The process safety time is the minimum time duration within which mitigation action to be completed by the controller to prevent the hazard from occurring [13]. The SDR QoS metric has been chosen here considering its mathematical simplicity for the purposes of demonstration. The approach is to illustrate the data analytics part of evaluation and simulation of other aspects such as channel characteristics, noise environment, protocol algorithms, etc., are not under the scope. This is because, the data analysis process illustrated here could be plugged-in with a communication model which may vary in respect of the said aspects for real time data processing. Further, optimization aspects of defense mechanisms to achieve the SIL target is part of design algorithm development and hence kept out of the scope for this simulation.

The simulation approach considers SIL-1 & 2 targets for demonstrating the proposed approach.

**Figure 2:**
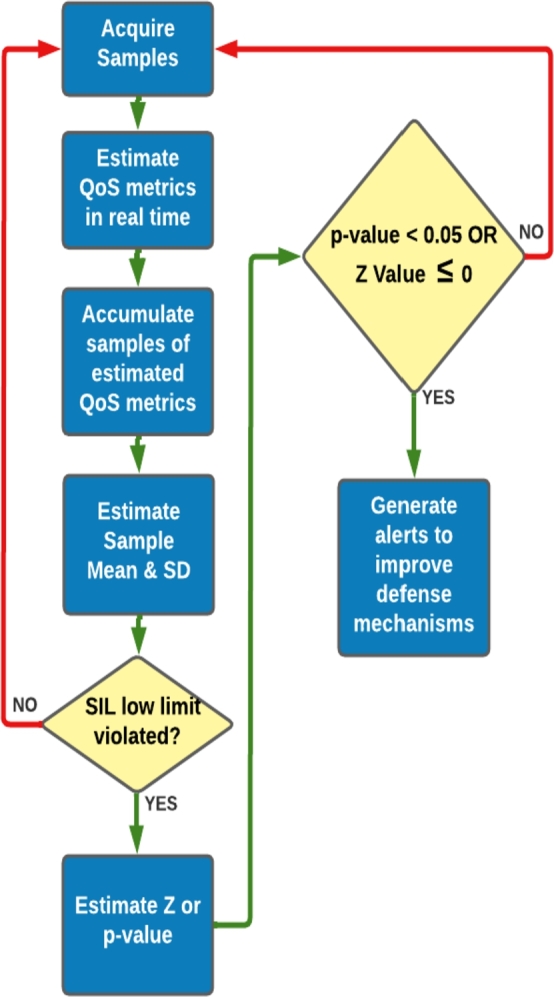
Data processing sequence for safety evaluation.

The SDR is a figure of merit and hence needs to be maximized for a safer system. The data processing sequence for safety evaluation is shown as a flow chart in Fig. 2. The simulation exercise has been carried out using MINITAB statistical software [37]. The SDR metric is simulated as a binomial random data and a total of 1 Million random data points were generated. Each of the data point is derived as an outcome of 25 trials. The binomial probability values chosen for the simulation and respective SIL target values are summarized in Table 6. From this population of 1,000,000 random points, a matrix of 2500 random samples were chosen and arranged into 50 rows and 50 columns. The rows & columns are designated with index ‘I’ and ‘J’ respectively. Each of the 50 sampled row elements are the simulated SDR cases for SIL1 target are referred as p_s1,_ p_s2_ and p_s3._ Where p indicates the probability chosen for simulation and s1, s2 and s3 refer to the simulation case 1, 2 and 3 respectively.

**Table 6:** SDR simulation test cases.

Safety Integrity Level	Low Demand System (probability of dangerous failure on Demand per guidelines)	Target SDR (1-probability of dangerous failure on Demand)	Simulated SDR Categories		
			(p_s1_)	(p_s2_)	(p_s3_)
SIL 1	≥10^−2^ to <10^−1^	0.9-0.99	0.93	0.95	0.97
SIL 2	≥10^−3^ to <10^−2^	0.99-0.999	0.993	0.995	0.997

For example, with SIL-1 as the target, the p_s1_ simulation case indicates the SDR probability of 0.93 for p_s2_ it is 0.95 and for p_s3_ it is 0.97. The corresponding sub-group means are referred as Mean_093, Mean_095 and the Mean_097 in the analysis. The mean of all subgroup means is designated as ‘*μ*’. The sampling serves the purpose of minimization of biasing error if any in the random number generator. Also, the sample size is chosen to be 50 for the sampling distribution to be near normal per central limit theorem. Similarly, for SIL2 target, the simulated SDR probabilities are 0.993, 0.995 and 0.997. The respective subgroup means are referred as Mean_0993, Mean_0995 and the Mean_0997.

**Figure 3:**
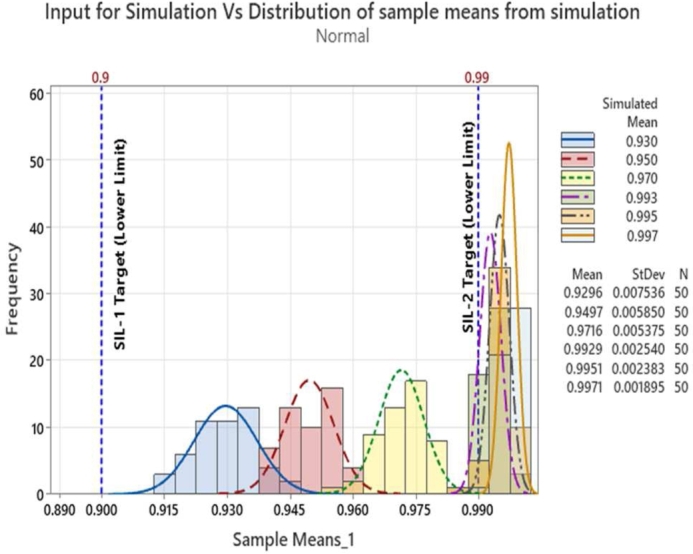
Input mean values for simulation vs sample mean from simulation.

**Figure 4:**
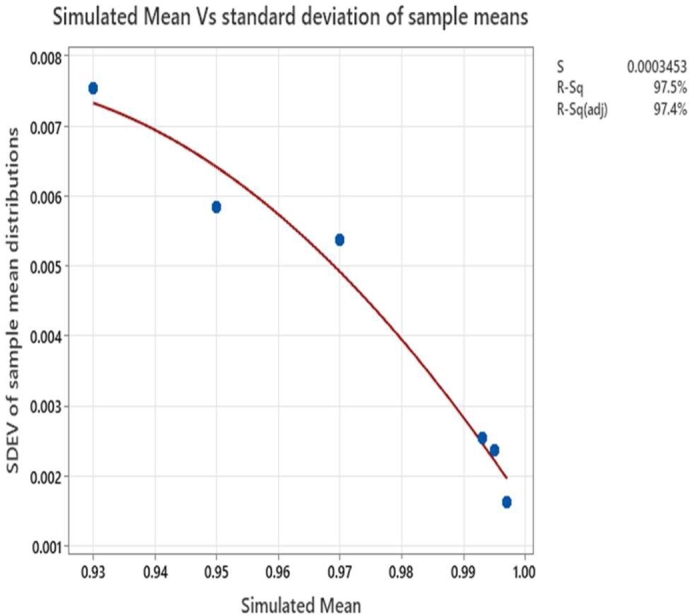
Input simulated mean values vs sample mean standard deviation.

The input mean values set for the simulation vs the sample mean obtained from the simulation is shown in Fig. 3. The graph indicates the coverage of input probability values during the simulation. We can observe at lower probability values the sample mean is higher than the minimum SIL-1 target whereas at higher probability values, the SIL-2 lower target values (0.99) are not met even though the standard deviation of the sample means at higher simulated mean values are lesser than that at lower simulated means. This variation is attributed to the random generator. The standard deviation for the sample means is shown in Fig. 4.

**Figure 5:**
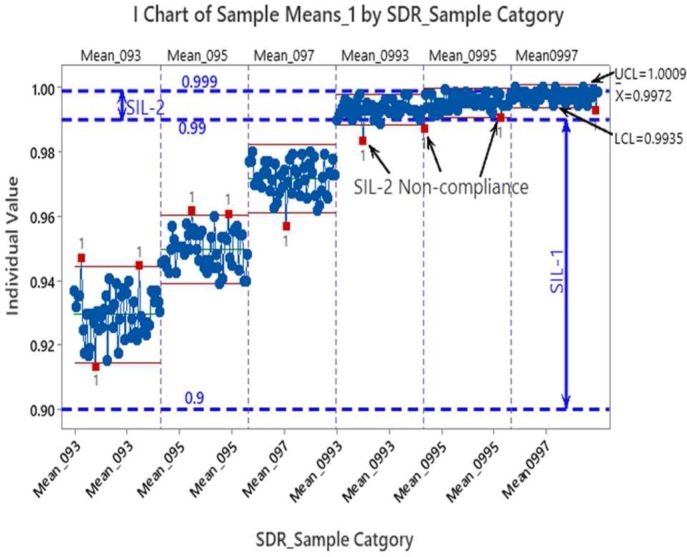
SDR mean control chart.

The sampling template is shown in Table 7. With the subgroup means collected for all the simulation cases per Table 6, a control chart is built to check the deviation of average SDR values from the SIL targets superimposed on the control chart. Here the objective is to assess if the mean value of SDRs can meet the SIL target values. The upper and lower control limits of the control chart (UCL, LCL) were respectively derived as (Mean + 3* Standard Deviation) and (Mean - 3* Standard Deviation). The standard deviation is based on the pooled standard deviation of the data i.e., samples in a subgroup. The control chart of all subgroups is shown in Fig. 5. Also shown here are the SIL1 and SIL2 probability limit lines. While the control limits in the chart help to assess if the communication process is under statistical control, the interest is more to assess if the observed mean SDR values are violating the minimum SIL target values. The red dots marked as ‘1’ in Fig. 5 signify the control chart rule number set to capture exceedances that cross the upper and lower control limits in a typical statistical process control chart.

**Table 7:** SDR sampling template.

Sample ID							
I↓∖J→	J = 1 to 50				Sub-group means	Mean of all subgroups	Standard Deviation of subgroup means

1					$\bar{X_{1J}}=\frac{\sum_{J=1}^{50}X_{1J}}{50}$	$\mu =\frac{\sum_{I=1}^{50}\bar{X_{IJ}}}{50}$	*S*_*Samples*_= $\sqrt{\sum_{n=1}^{50}\frac{{\left(\mu -{\bar{X}}_{IJ}\right)}^{2}}{50}}$
2					$\bar{X_{2J}}=\frac{\sum_{J=1}^{50}X_{2J}}{50}$		
:	:				:		
:	:				:		
:	:				:		
:	:				:		
50					$\bar{X_{50J}}=\frac{\sum_{J=1}^{50}X_{50J}}{50}$

As mentioned before not all these marked points are of our current focus. Since the SDR metric being a figure of merit, only the exceedance of the lower SIL target is of real safety concern for us (blue dotted lines). The Z and p values of subgroup means with respect to lower SIL target limits need to be captured to examine if the occurrence is statistically significant.

The exceedance captured as violating the upper control limit could be ignored as it surpasses the safety targets set which is a positive scenario. The statistically significant occurrences are different from random exceedances in that the former are caused by deterministic causes arising out of design aspects whereas the latter are due to random factors arising typically out of the operating environment. The deterministic causes are also referred as the special causes and typically include algorithm design, protocol features, protocol implementation methods, predictable noise characteristics of the channel etc., The random causes on the other hand are due to climatic changes, electromagnetic interferences etc. and such occurrences could be avoided by conditioning the work environment and appropriate process setup. The special cause induced occurrences can be mitigated by improving the communication defenses against the threats discussed before. The applicable defense mechanisms that need to be improved can be identified from the mapping matrix in step 4.

In the field application scenarios either of these metrics could be adopted. The Z-value is the number defined as shown in equation (2) and the corresponding p-value is derived by subtracting 0.5 from the cumulative probability value of the Z-value. The Z and p-values are used as the metrics to classify if the observed subgroup mean with respect to the lower SIL target value is statistically significant or otherwise. From equation (2) it can be observed that the Z-value decreases with increasing ‘$S_{SG}$’ the standard deviation of sub-group means.

A higher standard deviation of sub-group means indicates higher chances of violating the SIL lower target values and hence increasing possibility of non-compliance. Practically a higher ‘$S_{SG}$’ would occur when the wireless channel is affected by interference resulting in the communication threats discussed in Section 2. The numerator in equation (2) is the measure of gap between the subgroup mean i.e., the average value of QoS metric and the SIL target.

(2) $$Z_{IJ}=\frac{\left(\bar{X_{IJ}}-SILn_{LL}\right)}{S_{SG}}$$

In which

• $\bar{X_{IJ}}$ is the mean of I^th^ (row) subgroup having 50 columns of data
• $SILn_{LL}$ is the lower success probability limit of Safety Integrity Level- ‘n’
• $S_{SG}$ are the standard deviation of subgroup means

The p-value derived from equation (3) represents the probability that the QoS metric violates the lower SIL target limit due to random causes. Which means a higher p-value implies higher chances for the violation to be due to random causes whereas the lower p-value attributes the violation to special causes. The reason for subtracting 0.5 to derive the p-value is that it would yield -ve p-value when subgroup means are lower than lower SIL probability limit under consideration and makes the filtering algorithm comparatively simpler.

(3) $$p=\left\{ \begin{array}{cc} F{\left(X\right)}_{Z_{IJ}}-0.5,\; & Z_{IJ}\geq 0\\ 0,\; & Z_{IJ}<0 \end{array}\right.$$

In equation (3) $F{\left(X\right)}_{Z_{IJ}}$ is the cumulative probability from the normalized distribution for the given Z_IJ_.

**Figure 6:**
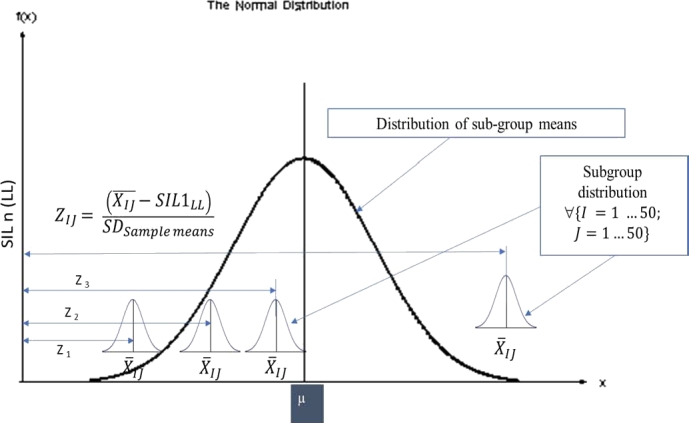
Subgroups and sample distribution.

**Figure 7:**
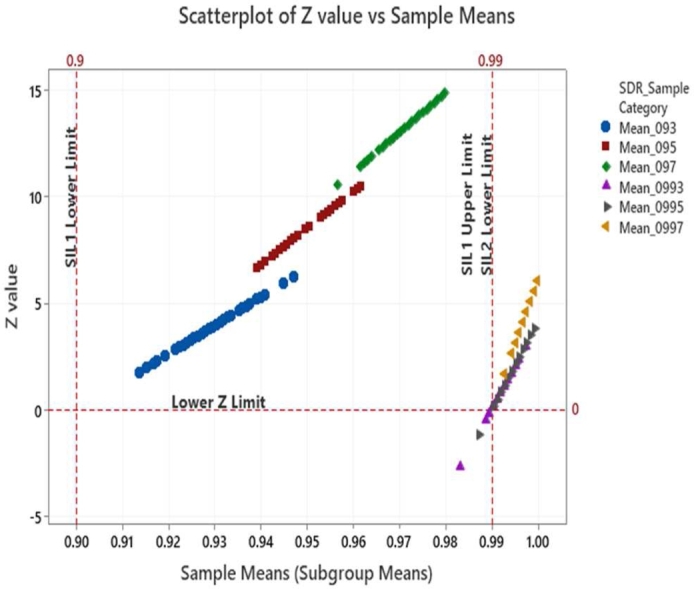
Z-values of sample means.

**Figure 8:**
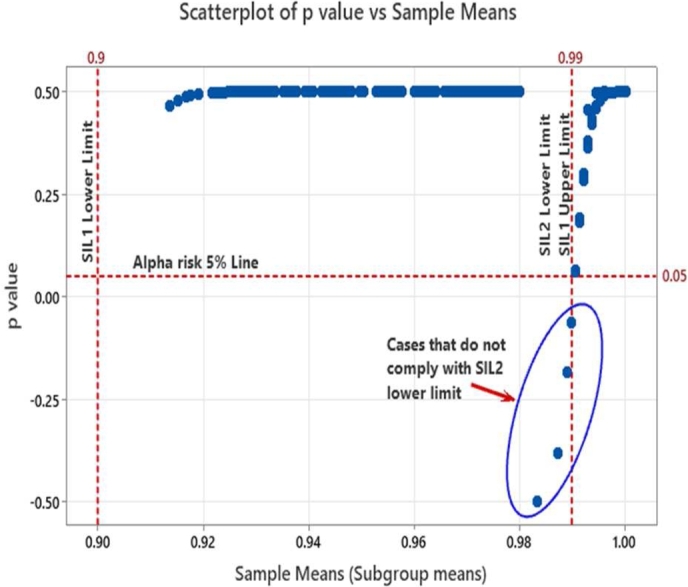
p-values of sample means.

From equation (2), it may be observed that when the sample mean $X_{IJ}$ becomes lower than the lower success probability limit of $SILn_{LL}$ then the $Z_{IJ}$ value will be negative, and the corresponding p-value becomes ‘0’ since -ve p-value has no significance. In our case the lower limits are respectively 0.9 and 0.99 corresponding to SIL-1 and SIL-2 requirements. When the sample mean becomes higher than the lower limit of $SILn_{LL}$, the numerator of equation (2) becomes positive and the Z value increases hence the corresponding p-value also will increase. This indicates better safety margin corresponding to the sample data w.r.t. to the lower limits of $SILn_{LL}$. One other possible case is when the sample mean is just equal to the lower $SILn_{LL}$ limits. Under such a condition the numerator of equation (2) will become zero and hence the p-value becomes zero indicating that the occurrence of sample mean is significant event and there is no safety margin. Under such scenarios design efforts should be considered for improving the safety margin using the communication defenses or investigating other possible special root causes if any. The sample means and distributions of sample means are illustrated in Fig. 6. The Z & p-values captured from our simulation results are provided as scatter plots in Fig. 7 and Fig. 8. Also superimposed are the lower limits of SIL-1 and SIL-2 as well as the minimum acceptable limits of Z and p values.

**Figure 9:**
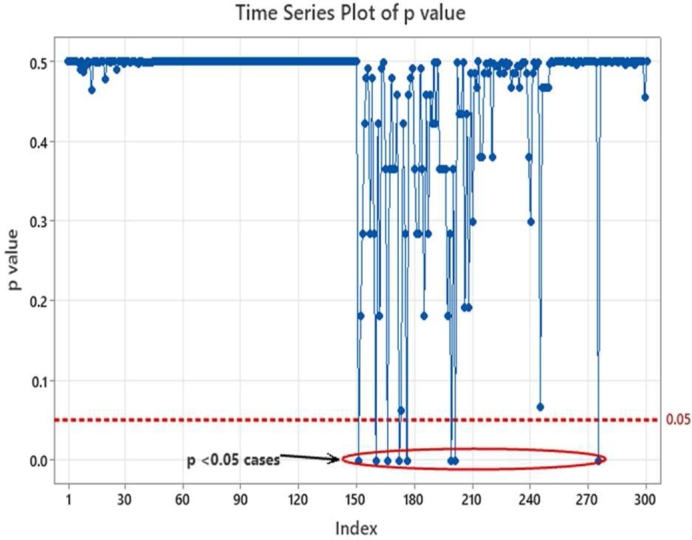
p-values of sample means over the time scale.

From Fig. 7 and Fig. 8 we find that the sample means from the simulated SDR categories of 0.93, 0.95 and 0.97 (referred as Mean_093, Mean_095 and Mean_097) are all falling within the lower and upper boundaries of SIL-1. Correspondingly the Z values of these points are above Z = 0 limit line and to the right of SIL-1 lower boundary line in Fig. 7. To account for an alpha risk of 5% in the decision process, i.e., categorizing the observed mean as violating the lower SIL limit value, p-values above 5% alpha risk line are considered meeting the SIL-1 lower probability boundary. This 5% margin builds a safety factor into the decision. In case of SDR categories belonging to SIL-2 probabilities such as 0.993, 0.995 and 0.997 (referred as Mean_0993, and mean_0997) while most of the data points are above the lower limit value of 0.99, non-compliances are observed from the Z-value and p-value plots in Fig. 7 and Fig. 8. The list of points that violate the SIL-2 lower limit values along with the Z and p-values are summarized in Table 8. The p-values over the time scale are indicated in Fig. 9. It was observed that the Z and p-values of these violating points are respectively <0 and <0.05 indicating that these are significant variations that are due to special causes and could be mitigated through design measures as discussed previously.

**Table 8:** Z & P-values of noncomplying cases.

SDR Sample Category (Sub-groups)	Sample Means $\bar{X_{IJ}}$	Sub-group Means	Sub-group SD $S_{SG}$	SIL Lower Limit $SILn_{LL}$	Z-Value $Z_{IJ}$	p-Value *p*	Valid p-value
Mean_0993	0.9896	0.9929	0.0025	0.99	−0.1575	−0.0626	0
Mean_0993	0.9896	0.9929	0.0025	0.99	−0.1575	−0.0626	0
Mean_0993	0.9832	0.9929	0.0025	0.99	−2.6770	−0.4963	0
Mean_0993	0.9896	0.9929	0.0025	0.99	−0.1575	−0.0626	0
Mean_0993	0.9888	0.9929	0.0025	0.99	−0.4724	−0.1817	0
Mean_0993	0.9896	0.9929	0.0025	0.99	−0.1575	−0.0626	0
Mean_0995	0.9872	0.9951	0.0024	0.99	−1.1751	−0.3800	0

Too frequent observation of p-values lesser than 0.05 over time signify sustained special cause induced violations of SIL lower limit and emphasizes the urgent need for the root cause analysis and corrective actions by improving the defenses.

## 7 Conclusions and future research opportunities

In this paper we have highlighted the upcoming trend of deploying WSN systems for industrial safety applications and discussed an approach to leverage the QoS metrics to assess the system Safety Integrity Level in the real time. We believe this methodology would help wireless network and IoT engineers to design and deploy WSN systems for industrial safety applications. Further to identify suitable QoS metrics for safety integrity assessment, we have also discussed a mapping technique that can relate the QoS metrics and communication defenses/safety mechanisms.

The mapping matrix can also benefit network safety engineers to determine safety mechanisms needed to improve the QoS metrics there by improving the safety integrity level of WSN system. We believe the safety assessment process and the simulation case example used to illustrate the approach would enable network safety design engineers to evolve the real-time algorithms to monitor the system safety integrity level. Finally, we would like to conclude by bringing out the following future research opportunities in this area for enhancing the safety integrity level of industrial WSN systems.

a) Develop real-time algorithm to evaluate compliance of WSN to functional safety goals SIL 1-4 based on QoS metrics

b) Develop prognostic QoS trend monitoring algorithms to serve as an additional layer of protection for the WSN safety system

c) Extend the QoS metrics-based safety assessment to adjacent areas such as automotive, medical electronics, unmanned aerial vehicles etc. considering respective safety integrity targets

d) Develop routing algorithms to maximize safety in WSN including the integrated WSN-MANETs.

e) Develop a channel model to further study the effect of defense mechanisms and its sensitivity to achieve overall End-to-End safety of WSN systems.

It is our considered viewpoint that functional safety is one of the critical elements especially in the context of rapidly evolving technologies like artificial intelligence, IoT, wireless, etc., for the application segments such as Industry 4.0, hospitals of future, smart buildings, autonomous vehicles, collaborative robots, etc. Hence, pursuing the future opportunities will enable creating an industrial environment that is functionally safer.

## Declarations

### Author contribution statement

Sivasubramanian Srinivasan: Conceived and designed the experiments; Performed the experiments; Analyzed and interpreted the data; Contributed reagents, materials, analysis tools or data; Wrote the paper.

Dr. T K Ramesh; Roberto Paccapeli; Luca Fanucci: Analyzed and interpreted the data.

### Funding statement

This work was supported by 10.13039/100009526Amrita Vishwa Vidyapeetham University.

### Data availability statement

No data was used for the research described in the article.

### Declaration of interests statement

The authors declare no conflict of interest.

### Additional information

No additional information is available for this paper.
